# Automated MR spectroscopy single voxel placement in suspected diffuse glioma based on tumor anatomy

**DOI:** 10.1093/noajnl/vdag093

**Published:** 2026-04-11

**Authors:** Saahil Chadha, Sarah M Jacobs, Tal Zeevi, Durga V Sritharan, Nazanin Maleki, Niklas Tillmanns, Jan Lost, David Weiss, Arman Avesta, Wondwossen Lerebo, Khaled Bousabarah, MingDe Lin, Fatima Memon, Sanjay Aneja, Mariam S Aboian

**Affiliations:** Department of Radiology and Biomedical Imaging, Yale School of Medicine, New Haven, Connecticut, USA; Department of Therapeutic Radiology, Yale School of Medicine, New Haven, Connecticut, USA; Department of Radiology and Biomedical Imaging, Yale School of Medicine, New Haven, Connecticut, USA; Center for Image Sciences, University Medical Center Utrecht, Utrecht, The Netherlands; Department of Radiology and Biomedical Imaging, Yale School of Medicine, New Haven, Connecticut, USA; Department of Therapeutic Radiology, Yale School of Medicine, New Haven, Connecticut, USA; Department of Radiology, Children’s Hospital of Philadelphia, Philadelphia, Pennsylvania, USA; Department of Radiology and Biomedical Imaging, Yale School of Medicine, New Haven, Connecticut, USA; Department of Radiology and Biomedical Imaging, Yale School of Medicine, New Haven, Connecticut, USA; Department of Radiology and Biomedical Imaging, Yale School of Medicine, New Haven, Connecticut, USA; Department of Radiology, Massachusetts General Hospital, Harvard Medical School, Boston, Massachusetts, USA; Department of Radiology, Children’s Hospital of Philadelphia, Philadelphia, Pennsylvania, USA; Visage Imaging GmbH, Berlin, Germany (K.B.); Department of Radiology and Biomedical Imaging, Yale School of Medicine, New Haven, Connecticut, USA; Visage Imaging Inc, San Diego, California, USA (M.D.L.); Department of Radiology and Imaging Sciences, Emory University, Atlanta, Georgia, USA (F.M.); Department of Therapeutic Radiology, Yale School of Medicine, New Haven, Connecticut, USA; Yale Cancer Center, New Haven, Connecticut, USA (S.A.); Department of Radiology, Children’s Hospital of Philadelphia, Philadelphia, Pennsylvania, USA; Department of Radiology, University of Pennsylvania Perelman School of Medicine, Philadelphia, Pennsylvania, USA

**Keywords:** artificial intelligence, glioma, magnetic resonance spectroscopy

## Abstract

**Background:**

Magnetic resonance spectroscopy (MRS) complements conventional MRI in brain tumor diagnosis by measuring metabolites associated with various molecular pathways. However, inconsistent and inaccurate voxel placement, due to limited experience and time constraints, has hindered its clinical use. There is a critical need for greater precision and efficiency in MRS voxel placement, which could be addressed by an algorithm leveraging tumor anatomy to facilitate the automation of this process. This pilot study aimed to design and validate an automated, AI-driven MRS voxel placement tool to evaluate suspected diffuse gliomas.

**Methods:**

In this pilot study, the MRS single voxel auto-placement algorithm utilizes tumor sub-compartment segmentation from a pre-trained deep learning model. Preprocessing involves co-registration and isotropic resampling of multiparametric MRI. The algorithm iteratively adjusts the position and rotation of an MRS voxel mask over the segmented images to maximize enhancing tumor core inclusion while excluding necrosis. Two board-certified neuroradiologists independently and blindly assessed algorithm- and clinically-placed MRS voxels (*n* = 14) using a Likert scale.

**Results:**

Fourteen cases of MRS single-voxel placement were evaluated (median age and interquartile range, 47 years [24-64 years]; male, 10 patients [71%]). Overall quality ratings of 4 or 5 were assigned to 75% of clinically placed voxels and 79% of algorithm-placed voxels, without any statistical difference (*P* = .56). Similarly, no statistically significant differences were observed in position ratings (*P* = .30) or rotation ratings (*P* = .51).

**Conclusions:**

No statistically significant differences were observed between algorithm- and clinically placed voxels, highlighting the promise of AI-based automated MRS single-voxel placement.

Key Points

- AI tumor segmentations enabled automated MRS voxel placement for suspected diffuse gliomas.
- No significant difference was observed between our algorithm’s placement and manual methods.

Importance of the Study

This study addresses a critical need in the clinical application of magnetic resonance spectroscopy (MRS) for diffuse glioma evaluation. Manual MRS voxel placement is time-consuming, prone to variability, and requires specialized expertise, limiting its widespread adoption in clinical practice. The artificial intelligence (AI)-based algorithm developed in this study automates voxel placement using tumor anatomy. Whereas prior studies relied primarily on T_2_-weighted fluid attenuated inversion recovery (FLAIR) imaging or targeted predefined brain regions, our algorithm leverages both FLAIR and T_1_-weighted contrast-enhanced (T_1_C+) sequences to incorporate insights from multiple tumor sub-compartments. Validation by expert neuroradiologists suggested that the algorithm’s voxel placement quality is similar to manual methods, indicating its potential for clinical use pending further evaluation. Automating voxel placement will improve consistency, save time, and allow for more reliable metabolic assessments. Future implications include enhancing longitudinal studies, facilitating post-treatment monitoring, and continuing optimization for broader clinical integration.

Magnetic resonance spectroscopy (MRS) is a non-invasive imaging technique that provides metabolic information about brain tissue, complementing the anatomic details obtained from conventional magnetic resonance imaging (MRI).^1^^,^^2^ With the 2016 World Health Organization (WHO) Classification of Tumors of the Central Nervous System transitioning from solely histology-based classification to incorporating molecular parameters,^3^ MRS has recaptured the interest of many clinicians and researchers as a technique for non-invasive molecular marker determination.^4-6^ The quantification of 2-hydroxyglutarate has gained particular attention for its potential in identifying isocitrate dehydrogenase mutations.^7^^, ^^8^ In the evaluation of diffuse gliomas broadly, MRS can aid in the differentiation of tumor tissue from non-neoplastic changes and provide insight into tumor grade and biology.^4^^, ^^9^

While the research applications of MRS are ever-growing, the implementation of MRS in clinical practice remains challenging. MRS is time-consuming and requires expertise in acquisition, processing, analysis, and interpretation.^10^^,^^11^ Although MRS acquisition time has markedly decreased from over 20 min to approximately 4 to 5 min per scan, adding this extra time to multi-sequence MRI protocols remains a major obstacle to clinical adoption.^12^ Furthermore, accurate MRS voxel placement is essential for capturing representative metabolic data from the region of interest, particularly in heterogeneous tumors, where areas of enhancing tumor, non-enhancing infiltrative regions, and necrosis often coexist. However, in clinical practice, manual MRS voxel placement is typically performed by technologists who rely on visual assessment of anatomical MRI sequences such as T_1_-weighted contrast-enhanced (T_1_C+) and T_2_-weighted fluid attenuated inversion recovery (FLAIR). This approach is time-consuming, subject to variability, and may not consistently optimize voxel positioning to capture the most diagnostically relevant metabolic information. Specialized training is required to avoid craniofacial structures, and necrotic tumor portions, especially common in glioblastomas, can result in poor MRS signal quality and lead to partial volume effects.^11^ Neuroradiologist confirmation of MRS voxel placement creates additional delays and renders MRS acquisition impractical for many clinical workflows. Thus, there is a critical need for a standardized and automated method for voxel placement to reduce inter-operator variability, ensure consistent data quality, and accelerate MRS acquisition.

Earlier investigation into automated MRS voxel placement has largely prioritized reproducible anatomical targeting, well-suited for studies of normal brain metabolism; however, these approaches have limited applicability in neuro-oncology, where lesions are heterogeneous in location and composition.^13-16^ Moreover, the few automated voxel placement methods studied in glioma populations have focused on a limited subset of tumor presentations and typically relied on a single imaging feature, underscoring the need for more comprehensive strategies.^17^^, ^^18^

The primary objective of this pilot study was to develop and validate a proof-of-concept automated MRS single voxel placement method. We developed an artificial intelligence (AI)-based algorithm guided by tumor anatomy, including FLAIR hyperintensity, enhancing tumor, and necrotic regions. The developed algorithm was validated through expert neuroradiologist assessment. We hypothesized that automated voxel placement would demonstrate non-inferior performance compared to manual placement.

## Methods

**Figure 1.**
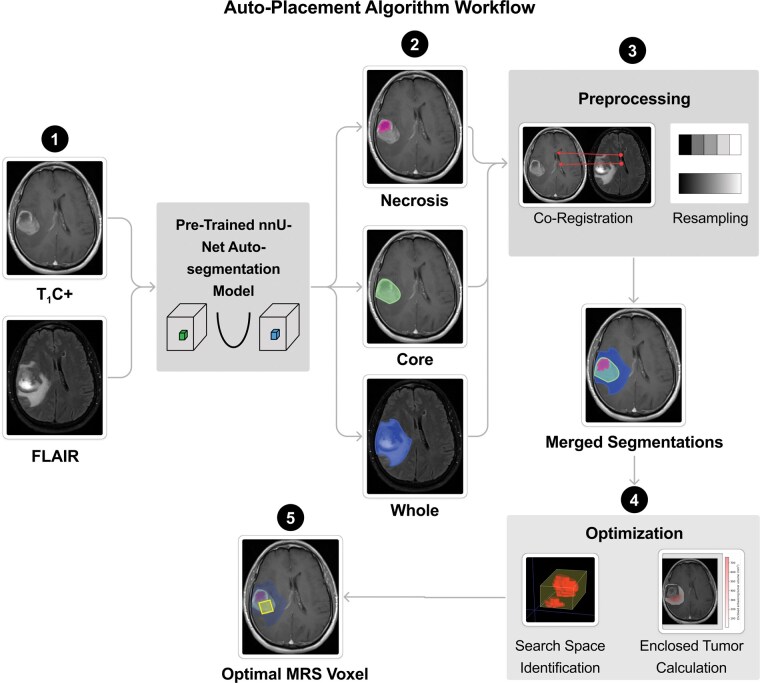
Auto-placement algorithm overview: (1) T_1_-weighted contrast-enhanced (T_1_C+) and fluid attenuated inversion recovery (FLAIR) images are input into a pre-trained nnU-Net model that (2) predicts enhancing core and necrosis on T_1_C+, as well as whole hyperintense tumor volume on FLAIR. (3) Images and segmentations are co-registered and resampled to a 1 mm isotropic voxel size. (4) The algorithm iterates over the search space around the enhancing core segmentation to identify the magnetic resonance spectroscopy (MRS) voxel orientation that maximizes enclosed core volume without necrosis, with whole peritumoral edema maximization as a tiebreaker. (5) The optimal MRS voxel orientation is output as a mask on T_1_C+ and/or FLAIR.

Our MRS single-voxel auto-placement algorithm utilizes the relevant pre-treatment imaging sequences, T_1_C+ and/or FLAIR, to determine lesion anatomy. Our approach was designed to accommodate the heterogeneity of both low-grade and high-grade gliomas; high-grade gliomas are more likely to exhibit tumor enhancement and necrosis, whereas low-grade gliomas often show minimal enhancement and lack necrotic regions. To account for these differences, a pre-trained auto-segmentation algorithm identifies and delineates up to three volumes of interest depending on the underlying tumor biology—enhancing core on T_1_C+, necrosis on T_1_C+, and the whole tumor volume defined by FLAIR hyperintensity. Using these imaging and segmentation data, the algorithm then identifies the optimal MRS voxel position and orientation, outputting this voxel as a mask on the input imaging. Figure 1 outlines the auto-placement algorithm workflow.

### Study Design and Cohort

This retrospective study was approved by the institutional review board with a waiver of informed consent. Pre-treatment MR imaging was retrieved from patients who underwent single-voxel proton MRS as part of the clinical workup for suspected diffuse glioma at the study institution between 1998 and 2023. MRS was performed to further characterize intra-axial lesions of uncertain etiology identified on MRI, providing metabolic information to aid diagnosis, guide biopsy planning, and help distinguish neoplastic from inflammatory or post-ictal processes. Patients were included in the validation cohort if they had available pre-treatment T_1_C+ and/or FLAIR MRI, archived single-voxel MRS spectra with documented clinically placed voxels, and adequate image quality without severe motion artifact. The cohort included both lower-grade and higher-grade gliomas. Clinically acquired MRS voxels were manually placed by trained MRI technologists and subsequently reviewed by board-certified neuroradiologists to confirm accurate targeting of the lesion or region of interest.

### MRS Acquisition

Single-voxel proton MRS was performed using either a spin echo sequence (90°-180°-180°) or a stimulated echo acquisition mode (STEAM) sequence (90°-90°-90°). Voxel size was fixed at 2 × 2 × 2 cm³. Scans were acquired using either a Siemens Verio or Trio scanner using standard clinical protocols. Detailed MRS acquisition parameters, including magnetic field strength, repetition time, and echo time, are summarized in Table 1.

**Table 1.** Patient demographics and image acquisition details for MRS cases used in Algorithm Quality Control Assessment

Characteristic	Summary
n	14
Age [years (IQR)]	47 (24-64)
Sex	
Male [*n* (%)]	10 (71%)
Female [*n* (%)]	4 (29%)
Histopathological diagnosis	
Oligodendroglioma [*n* (%)]	2 (14%)
Oligoastrocytoma [*n* (%)]	2 (14%)
Glioblastoma [*n* (%)]	2 (14%)
Astrocytoma [*n* (%)]	2 (14%)
Non-neoplastic [*n* (%)]	2 (14%)
Ganglioglioma [*n* (%)]	1 (7%)
Unknown [*n* (%)]	3 (21%)
Magnetic field strength	
3 T [*n* (%)]	13 (93%)
2.89 T [*n* (%)]	1 (7%)
Acquisition Parameters	
FLAIR TR [ms (IQR)]	5000 (5000-8750)
FLAIR TE [ms (IQR)]	395 (92-395)
T_1_C+ Gradient Echo TR [ms (IQR)]	1900 (1900-1900)
T_1_C+ Gradient Echo TE [ms (IQR)]	2.93 (2.93-3.07)
T_1_C+ Spin Echo TR [ms (IQR)]	541 (541-541)
T_1_C+ Spin Echo TE [ms (IQR)]	20 (20-20)
MRS Sequence	
Spin Echo [*n* (%)]	12 (85%)
STEAM [n (%)]	2 (15%)

STEAM indicates stimulated echo acquisition mode.

A copy of patients’ radiologic data was de-identified on the clinical production PACS (Visage 7, Visage Imaging, Inc., San Diego, CA) and sent to a research PACS instance (AI Accelerator, Visage Imaging, Inc.) for analysis. For each patient, both the original clinically-placed MRS voxel and an algorithm-generated voxel were overlaid on identical pre-treatment MR sequences and evaluated.

### Tumor Anatomy Determination

Tumor segmentation was performed using an nnU-Net^19^ model pre-trained on gliomas to identify regions of core enhancement and necrosis on T_1_C+, as well as areas of hyperintensity indicating whole tumor boundaries on FLAIR. This model was trained on a retrospective cohort of 397 patients with histopathologically-confirmed glioma and neuroradiologist-verified ground-truth segmentations. The first step of the proposed MRS single-voxel auto-placement algorithm uses this pre-trained nnU-Net model to generate anatomical segmentations on T_1_C+ and FLAIR for downstream analysis.

### Preprocessing

In cases that exhibited both contrast enhancement and FLAIR changes, typically high-grade gliomas, both the T_1_C+ and FLAIR sequence images were used as input for the auto-placement algorithm, along with their respective segmentations. The FLAIR image and whole tumor segmentation were co-registered onto the T_1_C+ image, aligning the images and segmentations within the same coordinate space. For cases that lacked contrast enhancement, typically low-grade gliomas, only the FLAIR image sequence was utilized, and the co-registration step was skipped. Subsequently, images were resampled to a 1 mm isotropic voxel size.

### Optimization

The auto-placement algorithm identified a 2 × 2 × 2 cm^3^ isotropic MRS voxel orientation, as used in clinical practice, that optimized the enclosed tumor portion. Because necrosis introduces noise into MRS spectra,^11^ in cases with enhancement on T_1_C+, the auto-placement algorithm maximized the enclosed enhancing tumor portion without enclosing any necrosis. In cases without enhancement, only the FLAIR image and whole segmentation were used as inputs. For these cases, the algorithm sought to maximize enclosed FLAIR hyperintensity.

In the first step of optimization, a search space was identified around the tumor. A bounding box was drawn around the enhancing tumor region. This bounding box was then padded by 2√3 cm—representing the limits for the center of the MRS voxel—and rotated from 0 to 90 degrees along the left/right, anterior/posterior, and superior/inferior axes. For each orientation tested, a 2 × 2 × 2 cm^3^ isotropic MRS voxel mask was generated on both T_1_C+ and FLAIR images, and intersected with the core, necrosis, and whole segmentations to determine the enclosed tumor volumes. After iterating over the search space of possible MRS voxel orientations, the algorithm output the optimal MRS voxel position and rotation as a mask on the input images.

### Expert Neuroradiologist Assessment

Two board-certified neuroradiologists (M.S.A. and F.M.) independently reviewed each case in a randomized, blinded fashion. Voxel placement was assessed using a 5-point Likert scale, where 1 represented poor quality and 5 represented strong quality. Cases were rated across three dimensions: voxel position, rotation, and overall placement quality. For each case, both the clinically placed and algorithm-placed voxels were presented to the readers in random order alongside the corresponding unmodified MR images. To ensure blinding, the images were formatted identically, with the same MR sequences, image slices, and resolution used for both voxel placements.

Median ratings from the two readers were used for group-level comparisons. A Wilcoxon signed-rank test was used to compare the Likert scores between clinically placed and algorithm-placed MRS voxels. Inter-rater differences were also assessed using a Wilcoxon signed-rank test comparing scores assigned by the raters. *P* < .05 was considered statistically significant.

### Statistical Analysis

Statistical analysis was conducted in Python (version 3.10). The statistics package of SciPy (version 1.11.2)^20^ was used to carry to carry out paired Wilcoxon signed-rank tests to compare ratings between algorithm placed and clinically placed voxels as well as between raters.

### Algorithm Implementation

The algorithm pipeline was written in Python (version 3.10). Image pre-processing was conducted using SimpleITK (version 2.2.1)^21^ and TorchIO (version 0.19.1).^22^ Optimization was conducted using TorchIO, SciPy (version 1.11.2),^20^ and NumPy (version 1.25.2).^23^ The segmentation and voxel optimization code are available at the following repository: https://github.com/ImagineQuant/mrs-voxel-placement.

## Results

### Pre-Trained Auto-Segmentation Model Training Cohort

The nnU-Net auto-segmentation model utilized in this study was pre-trained on a retrospective cohort of 397 patients. Of these, 35 patients (9%) had a 2021 WHO CNS grade 1 tumor, 97 patients (24%) had a grade 2 tumor, 53 (13%) had a grade 3 tumor, and 212 (53%) had a grade 4 tumor. Overall, the median age was 52 years (interquartile range [IQR], 33-65 years), and 241 patients (61%) were male. Imaging from 381 patients (96%) displayed hyperintensity on FLAIR, and 352 patients (89%) displayed enhancement on T_1_C+. Table 2 provides the baseline demographic and image acquisition details for these patients.

**Table 2.** Patient demographics and image acquisition details for glioma cases used to train the nnU-Net segmentation model

	WHO Grade				
Characteristic	1	2	3	4	Total
n (%)	35 (9%)	97 (24%)	53 (13%)	212 (53%)	397
Age [years (IQR)]	14 (4-21)	34 (24-47)	40 (36-60)	62 (52-71)	52 (33-65)
Sex					
Male [*n* (%)]	18 (51%)	53 (55%)	35 (66%)	135 (64%)	241 (61%)
Female [*n* (%)]	17 (49%)	44 (45%)	18 (34%)	77 (36%)	156 (39%)
Magnetic Field Strength					
3 T [*n* (%)]	18 (51%)	43 (45%)	27 (52%)	134 (64%)	222 (56%)
1.5 T [*n* (%)]	17 (49%)	49 (52%)	25 (48%)	77 (36%)	168 (43%)
Other [*n* (%)]	0 (0%)	3 (3%)	0 (0%)	0 (0%)	3 (1%)
FLAIR hyperintensity present [*n* (%)]	35 (100%)	85 (88%)	50 (94%)	211 (100%)	381 (96%)
Enhancement present [*n* (%)]	32 (91%)	70 (72%)	44 (83%)	206 (97%)	352 (89%)
Necrosis present [*n* (%)]	21 (60%)	26 (27%)	23 (43%)	194 (92%)	264 (66%)
Acquisition Parameters [*n* (%)]					
FLAIR TR [ms (IQR)]	9000 (8002-9113)	9000 (5000-9000)	9000 (9000-9000)	9000 (5000-9000)	9000 (8000-9000)
FLAIR TE [ms (IQR)]	127 (88-160)	142 (92-374)	93 (88-125)	92 (88-307)	97 (88-160)
T_1_C+ Gradient Echo TR [ms (IQR)]	500 (443-541)	490 (450541)	537 (474600)	541 (500592)	534 (468-552)
T_1_C+ Gradient Echo TE [ms (IQR)]	20 (16-20)	20 (12-20)	17 (11-20)	12 (10-20)	17 (11-20)
T_1_C+ Spin Echo TR [ms (IQR)]	1900 (1800-1900)	1900 (1700-1900)	1900 (1900-1900)	1900 (1900-1900)	1900 (1900-1900)
T_1_C+ Spin Echo TE [ms (IQR)]	2.96 (2.85-3.09)	2.93 (2.74-3.08)	2.95 (2.72-3.08)	2.98 (2.93-3.08)	2.96 (2.93-3.08)

### Validation Cohort Characteristics

Fourteen patients met the inclusion criteria and were included in the blinded expert neuroradiologist quality control assessment (Table 1). Tumor grade distribution was not proportional but reflects the actual distribution of cases that underwent MRS at our institution during the study period. Of the 14 patients, 2 patients (14%) had histopathological diagnoses of each of the following: oligodendroglioma, oligoastrocytoma, glioblastoma, and astrocytoma. Histopathological diagnoses for 2 patients (14%) were non-neoplastic, 1 was ganglioglioma (7%), and 3 were unknown (21%). The median age was 47 years (IQR, 24-64 years), and 10 patients (71%) were male. Of the 14 scans, 13 (93%) were acquired on a Siemens Verio and 1 (7%) on a Siemens Trio scanner. 12 scans (86%) used a spin echo sequence, and two (14%) used the STEAM sequence. Table 1 provides additional details on patient demographics, anatomical image acquisition parameters, and MRS pulse sequences. Spectroscopic acquisition parameters were not archived.

### Automated Voxel Optimization and Placement

**Figure 2.**
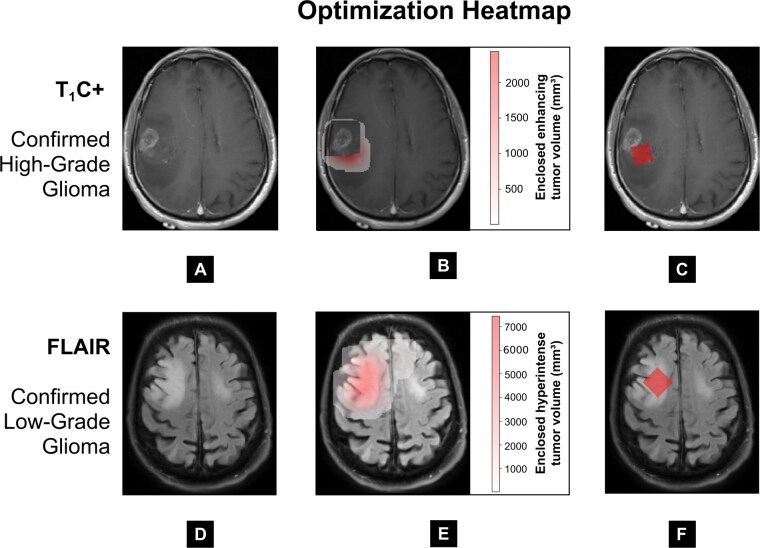
Optimization heatmap: (A, D) Input images of confirmed high-grade glioma on a T1-weighted contrast-enhanced (T1C+) image and confirmed low-grade glioma on a fluid attenuated inversion recovery (FLAIR) image, respectively. (B, E) Heatmaps where color corresponds to the enclosed core/whole segmentation volume for a potential 2 × 2 × 2 cm³ magnetic resonance spectroscopy (MRS) voxel centered at the given position. MRS voxels placed outside the search space and those that would intersect necrosis are left uncolored (B). (C, F) Optimal MRS voxel output masks in correspondence with the heatmaps.

**Figure 3.**
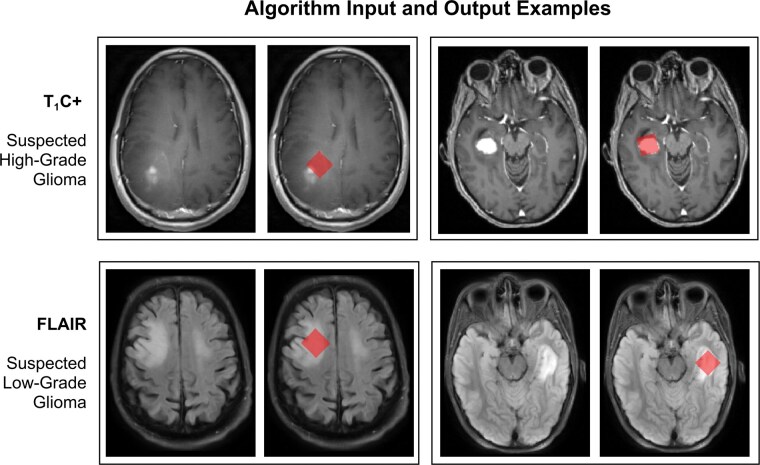
Algorithm input and output examples: Image pairs used in quality assessment. The unmodified T1-weighted contrast-enhanced (T1C+) and fluid attenuated inversion recovery (FLAIR) images are on the left of each pair, and the same image with optimal magnetic resonance spectroscopy (MRS) voxel masks output by the algorithm are on the right. The MRS voxel images were presented in random order with identical image formatting, resolution, and slice number. (Top) Cases demonstrating enhancement on T1C+. (Bottom) Suspected low-grade gliomas with only hyperintensity on FLAIR.

To manually validate the optimization process, heatmaps were generated. In Figure 2B, the heatmap is color-coded based on the volume of enhancing tumor enclosed when the MRS voxel is centered at each position in the image. Voxels located outside the search space or those enclosing necrotic tissue are excluded. Figure 2C shows the MRS voxel identified as the optimal solution after applying the optimization heuristic across all eligible voxel orientations. An example heatmap for a non-enhancing glioma is provided in Figure 2E. In this case, the optimization heuristic maximizes the enclosed FLAIR hyperintense tumor volume. Figure 3 shows additional examples of algorithm-placed MRS voxels.

### Expert Neuroradiologist Quality Assessment

**Figure 4.**
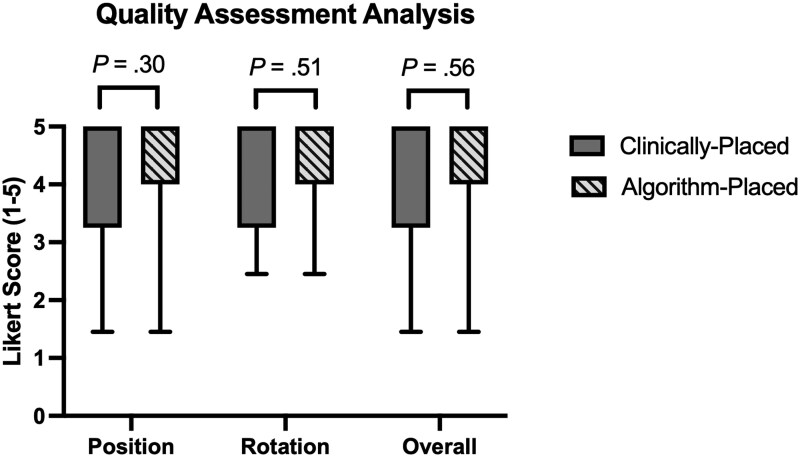
Quality assessment analysis: In a blinded study, two board-certified neuroradiologists rated position, rotation and overall placement of clinically placed voxels versus algorithm-placed voxels on a 5-point Likert scale, with 1 representing poor placement and 5 representing strong placement. Boxes indicate interquartile range, and whiskers indicate 5-95 percentile values. *P* values were calculated using Wilcoxon signed-rank tests.

Neuroradiologists rated 75% of clinically placed voxels with position scores of 4 or 5, compared to 82% of algorithm-placed voxels (*P* = .30). For rotation scores, 79% of clinically placed voxels were rated as a 4 or 5, compared to 82% of algorithm-placed voxels (*P* = .51). Finally, 75% of clinically placed voxels were given an overall rating of 4 or 5, compared to 79% of algorithm-placed voxels (*P* = .56). Figure 4 summarizes the results from this performance analysis, and Supplementary Table S1 provides all ratings by case. No significant inter-rater differences were observed during pooled analysis (*P* = .50), nor when separately analyzing position (*P* = .62), rotation (*P* = .45), and overall ratings (*P* = .97).

## Discussion

MRS is a powerful non-invasive tool for evaluating diffuse gliomas, providing valuable metabolic information that can aid in tumor characterization and treatment planning.^4^^,^^9^ Despite its potential, clinical application remains limited by the expertise and time required for accurate voxel placement. To address this challenge, we present a novel AI-driven automated MRS single-voxel placement algorithm based on tumor anatomy, which demonstrated placement quality comparable to that of experienced clinicians upon expert review.

In this pilot study, we applied an AI model to multiparametric MRI to segment key anatomical tumor sub-compartments, including enhancing tumor core, necrosis, and whole FLAIR hyperintensity. Using these segmentations, our algorithm systematically explores possible MRS voxel orientations to identify the optimal placement. The algorithm’s output is designed to be easily understood by clinicians and includes a heatmap feature for interpretability. In a blinded quality control assessment with expert neuroradiologists, 79% of algorithm-placed voxels received an overall rating of 4 or 5, compared to 75% for clinically placed voxels, with no statistically significant differences in voxel positioning, rotation, or overall placement quality. These findings suggest that the performance of our auto-placement algorithm is broadly comparable to manual placement and highlight its potential to support clinical voxel positioning, pending further validation.

Prior methods for automated MRS voxel placement have largely focused on ensuring consistent neuroanatomical positioning, often targeting predefined brain regions for metabolic analysis.^13-16^ However, these approaches are poorly suited for neuro-oncologic applications, due to the heterogeneity in the location and morphology of intracranial neoplasms. Previous studies applying automated MRS voxel placement in glioma patients have focused on low-grade diffuse gliomas and relied solely on FLAIR hyperintensity for guidance.^17^^,^^18^ In contrast, our approach offers the unique advantage of incorporating both FLAIR and T_1_C+ imaging, enabling the algorithm to capture tumor core while excluding necrotic regions—features that are especially critical for high-grade gliomas, like glioblastoma. This strategy has been shown to reduce partial volume effects and minimize noise and artifact.^11^ Importantly, unlike previous work, our study also includes clinical validation through a blinded quality control assessment involving board-certified neuroradiologists, confirming the algorithm’s strong performance.

This study has certain limitations. Though T_1_C+ imaging provides valuable information about tumor enhancement and necrosis, this sequence may be unavailable if MRS spectra are acquired before contrast administration. To address this, the developed algorithm is highly flexible and can operate using only FLAIR pre-contrast imaging or using patients’ prior contrast-enhanced MRI studies. Additionally, while varying the voxel size parameter may enable further optimization of spectral quality, we used a standard voxel size of 2 × 2 × 2 cm^3^, the most common in clinical practice, ensuring compatibility and promoting the integrity of our blinded quality control assessment. Given the retrospective nature of the dataset, acquisitions were performed across different scanner models, which may introduce variability in image characteristics and spectral quality; however, this is representative of real-world clinical heterogeneity. While voxel placement quality was assessed by blinded readers, subtle reader bias cannot be fully excluded despite the absence of systematic inter-rater differences. Finally, we acknowledge that other tissues, such as the ventricles and calvarium, can introduce noise into MRS spectra, and future optimizations can consider strategies for avoiding these regions.

Finally, as the first step in this proof-of-concept study, model development was restricted to single-voxel MRS acquisitions. Although multivoxel MRS was performed in many patients and often acquired alongside single-voxel MRS, we intentionally focused on single-voxel spectroscopy to establish feasibility using a standardized voxel geometry. Multivoxel MRS introduces additional heterogeneity related to spatial coverage and shimming that would require a distinct modeling approach.^24^ Extending this approach to multivoxel MRS represents an important direction for future work.

Future studies will focus on prospective validation in larger cohorts adopting quantitative metrics, such as spatial overlap and spectral quality such as signal-to-noise ratio, in addition to blinded neuroradiologist assessment. In this retrospective study, orientation metadata for clinically placed voxels were not consistently available, so voxel placement quality was assessed by expert review. Prospective studies with standardized capture of voxel geometry will enable direct spatial comparisons between algorithm-guided and clinically placed voxels. Beyond pre-treatment tumor characterization, MRS can also be used longitudinally, and a significant advantage of our algorithm is in its potential to facilitate assessment across serial imaging. By storing previous voxel placements, our algorithm could be used to ensure consistency across study time points. To expand its application in post-treatment glioma patients, future work will also focus on resection cavity segmentation and exclusion. For clinical translation, the algorithm could be integrated into PACS to display optimal voxel positions on structural MRI prior to MRS and ultimately into scanner prescriptive systems to provide real-time guidance for technologists at the point of care.

In summary, our automated MRS single-voxel placement algorithm has the potential to assist clinicians in placing voxels in orientations that optimize spectral quality. The performance of this approach has been validated through an expert quality control assessment, suggesting promise for clinical application. Future work will focus on additional feature development and prospective validation to further define its role in clinical practice.

## Supplementary Material

vdag093_Supplementary_Data

## Data Availability

Data used for segmentation model training and auto-placement algorithm evaluation are available upon request to the corresponding author.
