# Supplementary material for: Automated MR spectroscopy single voxel placement in suspected diffuse glioma based on tumor anatomy
Source: Neurooncol Adv. 2026 Apr 11;8(1):vdag093. doi: 10.1093/noajnl/vdag093 (PMC13161565; doi:10.1093/noajnl/vdag093)
Supplement: vdag093_Supplementary_Data [file vdag093_supplementary_data.docx]

|  |  | Position | | Rotation | | Overall | |
| --- | --- | --- | --- | --- | --- | --- | --- |
| Rater | **Case** | **Clinical** | **Algorithm** | **Clinical** | **Algorithm** | **Clinical** | **Algorithm** |
| 1 | 1 | 4 | 5 | 4 | 5 | 4 | 5 |
|  | 2 | 3 | 4 | 3 | 5 | 3 | 4 |
|  | 3 | 5 | 4 | 5 | 5 | 5 | 4 |
|  | 4 | 5 | 5 | 5 | 5 | 5 | 5 |
|  | 5 | 5 | 5 | 5 | 5 | 5 | 5 |
|  | 6 | 3 | 5 | 3 | 5 | 3 | 5 |
|  | 7 | 3 | 5 | 3 | 5 | 3 | 5 |
|  | 8 | 5 | 4 | 5 | 4 | 5 | 4 |
|  | 9 | 4 | 5 | 3 | 5 | 4 | 5 |
|  | 10 | 5 | 3 | 5 | 3 | 5 | 3 |
|  | 11 | 2 | 5 | 2 | 5 | 2 | 5 |
|  | 12 | 3 | 5 | 3 | 5 | 3 | 5 |
|  | 13 | 5 | 5 | 5 | 5 | 5 | 5 |
|  | 14 | 5 | 5 | 5 | 5 | 5 | 5 |
| 2 | 1 | 5 | 5 | 5 | 4 | 5 | 5 |
|  | 2 | 5 | 5 | 5 | 5 | 5 | 5 |
|  | 3 | 5 | 5 | 5 | 5 | 5 | 5 |
|  | 4 | 5 | 5 | 5 | 5 | 5 | 5 |
|  | 5 | 5 | 5 | 5 | 5 | 5 | 5 |
|  | 6 | 5 | 5 | 4 | 5 | 5 | 5 |
|  | 7 | 2 | 5 | 5 | 5 | 2 | 5 |
|  | 8 | 5 | 4 | 5 | 4 | 5 | 4 |
|  | 9 | 5 | 5 | 5 | 4 | 5 | 4 |
|  | 10 | 4 | 4 | 5 | 2 | 4 | 2 |
|  | 11 | 1 | 1 | 3 | 3 | 1 | 1 |
|  | 12 | 5 | 5 | 5 | 5 | 5 | 5 |
|  | 13 | 5 | 2 | 5 | 3 | 5 | 2 |
|  | 14 | 5 | 5 | 5 | 5 | 5 | 5 |

**Table S1. Ratings from Expert Neuroradiologist Quality Assessment.** Algorithm- and clinically-placed voxels were rated on a 5-point Likert scale (1 representing poor placement and 5 representing strong placement). Two expert neuroradiologists rated voxel position, rotation, and overall placement across 14 cases.
